# Mutational dynamics of the SARS coronavirus in cell culture and human populations isolated in 2003

**DOI:** 10.1186/1471-2334-4-32

**Published:** 2004-09-06

**Authors:** Vinsensius B Vega, Yijun Ruan, Jianjun Liu, Wah Heng Lee, Chia Lin Wei, Su Yun Se-Thoe, Kin Fai Tang, Tao Zhang, Prasanna R Kolatkar, Eng Eong Ooi, Ai Ee Ling, Lawrence W Stanton, Philip M Long, Edison T Liu

**Affiliations:** 1 Genome Institute of Singapore, 60 Biopolis Street, Singapore 138672; 2 Virology Section, Department of Pathology, Singapore General Hospital, Singapore; 3 Environmental Health Institute, 41 Science Park Road, Singapore Science Park II, Singapore 117610; 4 Center for Computational Learning Systems, Columbia University, New York, NY 10027 USA

## Abstract

**Background:**

The SARS coronavirus is the etiologic agent for the epidemic of the Severe Acute Respiratory Syndrome. The recent emergence of this new pathogen, the careful tracing of its transmission patterns, and the ability to propagate in culture allows the exploration of the mutational dynamics of the SARS-CoV in human populations.

**Methods:**

We sequenced complete SARS-CoV genomes taken from primary human tissues (SIN3408, SIN3725V, SIN3765V), cultured isolates (SIN848, SIN846, SIN842, SIN845, SIN847, SIN849, SIN850, SIN852, SIN3408L), and five consecutive Vero cell passages (SIN2774_P1, SIN2774_P2, SIN2774_P3, SIN2774_P4, SIN2774_P5) arising from SIN2774 isolate. These represented individual patient samples, serial in vitro passages in cell culture, and paired human and cell culture isolates. Employing a refined mutation filtering scheme and constant mutation rate model, the mutation rates were estimated and the possible date of emergence was calculated. Phylogenetic analysis was used to uncover molecular relationships between the isolates.

**Results:**

Close examination of whole genome sequence of 54 SARS-CoV isolates identified before 14^th ^October 2003, including 22 from patients in Singapore, revealed the mutations engendered during human-to-Vero and Vero-to-human transmission as well as in multiple Vero cell passages in order to refine our analysis of human-to-human transmission. Though co-infection by different quasipecies in individual tissue samples is observed, the in vitro mutation rate of the SARS-CoV in Vero cell passage is negligible. The in vivo mutation rate, however, is consistent with estimates of other RNA viruses at approximately 5.7 × 10^-6 ^nucleotide substitutions per site per day (0.17 mutations per genome per day), or two mutations per human passage (adjusted R-square = 0.4014). Using the immediate Hotel M contact isolates as roots, we observed that the SARS epidemic has generated four major genetic groups that are geographically associated: two Singapore isolates, one Taiwan isolate, and one North China isolate which appears most closely related to the putative SARS-CoV isolated from a palm civet. Non-synonymous mutations are centered in non-essential ORFs especially in structural and antigenic genes such as the S and M proteins, but these mutations did not distinguish the geographical groupings. However, no non-synonymous mutations were found in the 3CLpro and the polymerase genes.

**Conclusions:**

Our results show that the SARS-CoV is well adapted to growth in culture and did not appear to undergo specific selection in human populations. We further assessed that the putative origin of the SARS epidemic was in late October 2002 which is consistent with a recent estimate using cases from China. The greater sequence divergence in the structural and antigenic proteins and consistent deletions in the 3' – most portion of the viral genome suggest that certain selection pressures are interacting with the functional nature of these validated and putative ORFs.

## Background

The Severe Acute Respiratory Syndrome (SARS) was first reported in November 2002 and rapidly spread to a number of distant global regions by early 2003. A new coronavirus, the SARS-CoV, was identified to be the cause of SARS [1,2] and was rapidly sequenced and characterized [3,4]. SARS-CoV is an enveloped, positive strand RNA virus with a wide host range. Recombination and mutation rates of RNA viruses are high, several orders of magnitude higher than DNA based microbes and in eukaryotes, and have been the cause of rapid changes in antigenicity, virulence, and drug sensitivity. Thus, the direct estimate of the mutation rates of the SARS-CoV in human populations and the analysis of the mutational spectrum would aid in developing strategies for monitoring and therapy.

Previously, our analysis of 14 SARS sequences (five of which originated from Singapore) in May 2003 indicated that there are two different genotypes circulating in the world [5]. Recently, there has been a substantial increase in the number of SARS-CoV genomes sequenced. A total of 54 SARS-CoV genomic sequences (37 from the public database prior to October 14, 2003 and 17 sequenced within our institute) are used in our current analysis. This large dataset coupled with the availability of clinical data for cases related to Singapore patients and our molecular observations during in vitro cell passage presents an opportunity for a comprehensive analysis of the SARS-CoV mutational behavior.

## Methods

### Viral RNA genome isolation and sequencing

SARS-CoV from the primary patient tissues were isolated by homogenizing the tissues in PBS buffer followed by a low speed centrifugation to obtain the viral particle containing supernatant. The virus-containing samples were also inoculated into Vero cell E6. The cells were maintained at 37°C using the usual viral cell culture media, and repassaged after 7 days of incubation. The virus-containing supernatants of homogenize or different passages of Vero cell E6 showing CPE were centrifuged at 23,000 RCF for 2.5 hours to pellet the viral particles and followed by RNA extraction using the QiAmp viral RNA mini kit (Qiagen, ). The RNA genome templates were converted into double strand cDNA and sequenced as previously described [5]. The processing of raw sequence reads (base calling, assembly, and editing) was done using PHRED/PHRAP/CONSED (University of Washington, Seattle, WA, USA, ).

### Genotype determination using MassArray technology

A number of single nucleotide variations (SNVs) were further confirmed using a sensitive Mass Spectrometry based genotyping assay that was developed within our institute [6]. The RNA of the virus was first isolated using QiAmp viral RNA mini kit and then reverse-transcribed into cDNA (using the RNA as template, SuperScript kit from Invitrogen, and sequence specific primers), which were further purified. Primer extension assays were carried out for the SNVs of interest. The extension products were then detected in the MassARRAY (from Sequenom) to determine the genotypes.

### Data and statistical analysis

We aligned the 54 SARS-CoV genomes using CLUSTALW [7]. To minimize the effect of sequencing errors and other artefacts to our analysis, we employed a filtering scheme where only SNVs shared by more than two different isolates are kept. The phylogenetic trees were reconstructed using the filtered variations. The reconstruction was done using PAUP* [8] with Maximum Likelihood criterion, keeping the other parameters to the default.

The significance of the variations that pass the proposed mutation filter (where only mutations shared by more than 2 out of 54 isolates are considered real) can be assessed by calculating the probability that a random noise would meet the filtering criterion. The null hypothesis is that the noisy variations are generated independently between genomes. Let *q *be the rate of noisy mutation in a genome (based on our findings, as reported earlier in the text, we conservatively set 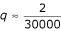, i.e. about two per SARS-CoV genome). The probability that, at a given nucleotide, a noisy mutation is shared by exactly *i *out of *m *different isolates is 
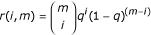
. Thus, the probability that a given nucleotide position has an erroneous mutation shared by more than *k *isolates is 
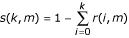
. In a genome with *n *bases, applying the Bonferroni inequalities, the probability that at least one position is corrupted by noise more than *k *times is *p*(*k*,*n*,*m*) ≤ *n *× *s*(*k*,*m*). In the case of 54 SARS-CoV genomes analyzed in this paper, 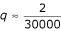, *m *= 54, *k *= 2, *n *= 30000, and hence the *p*-value of mutations that satisfy the filter is ≤ 2.2 × 10^-4^.

In the estimation of SARS-CoV daily mutation rate, we employed the conservative constant mutation rate model [9], where the number of mutations *d *found in an isolate from its ancestor is proportional to the mutation rate *k *and the temporal difference *t *between the isolate and its ancestor, so that *d *= *k *× *t*. Based on the Singapore patients contact tracing information (see Figure 4), we obtained 6 pairs of isolates with known definite ancestor-descendant relationship, calculated the number of mutations (*d*) and the time difference (*t*) for each pair, and estimate the mutation rate *k *for the model using least square fitting. The goodness-of-fit were measured using the adjusted R-square statistics.

Another pertinent question in the analysis of SARS-CoV evolution is prediction of the possible date of origin of the human SARS-CoV. Based on the animal-origin hypothesis of SARS-CoV, we assumed the SARS-CoV isolated from palm civet cat as the putative principal isolate that infected the human population. Adhering to the constant mutation rate model, we fit the following model: *d*_*x *_= *d*_0 _+ *kx*, where *k *is the daily rate of mutation, *x *is the sampling date measured relative to 1^st ^November 2002, and *d*_*x *_is the number of mutations, as compared to the civet cat isolate, of the isolate sampled at date *x*. Twelve data points were calculated and used to fit the model. The date of origin can be solved by solving *x *for *d*_*x *_= 0. The goodness-of-fit was measured using the adjusted R-square statistic.

## Results and discussion

### SARS-CoV mutations *in vitro*

First, we sought to determine the rate of mutation of the SARS-CoV in Vero cell culture. To achieve this, we subjected SIN2774 isolate to 5 passages in Vero cells. At the appearance of cytolysis at each passage samples were withdrawn and their genomes completely sequenced. Any ambiguities by capillary sequencing were clarified by mass spectroscopic validation [6]. Our results showed that the Vero cell passages are actually comprised of two coexisting quasispecies bearing either an A or G at position 18372. No mutations emerged upon passage, and the ratio of A/G at 18372 remained constant over the passages (Table 1). This suggests that the mutation rate in culture of SARS-CoV is very low at <1 in 5 passages. Thus artificial mutations from limited *in vitro *cell culture are negligible.

### Mutations associated with human-to-Vero and Vero-to-human transition

Next, we asked whether the transition from human tissue to growth in Vero cell culture engendered either mutations or clonal selection. The SARS-CoV were sequenced from three human tissue-Vero cell culture pairs of viral samples from Singapore and one pair was obtained from the public domain (see supplemental information, Table S1). The results shown in Table 2 showed that mutations emerged in only one case of human to Vero cell passage posted in Genbank (AS → HSR1) but in none of the Singapore pairs. However, the viral sequence from SIN3725V isolated from a lung sample showed evidence for co-infection by two distinct genotypes of SARS CoV. This was manifested by the simultaneous presence of T and C at positions 548, 1727, 13347, confirmed by genotyping using MALDI-TOF MassARRAY technology. Subsequent deconvolution by tracing the "haplotypes" at these loci in different Singaporean isolates revealed probable sequence signatures of T, T, C at these locations in one isolate and C, C, T in the other (see Table S2). Though tissue-derived SIN3725V has two SARS-CoV quasispecies, the isolate after subsequent Vero cell culture showed only one (bearing the T, T, C haplotype, supplemental information, Table S2). These results again show that coinfection by multiple quasispecies is not uncommon in human tissues, and that passage to Vero cells may either generate new mutations at a low rate, or titrates out one quasispecies in the transition.

Singapore encountered an unusual incident where a stable lab SARS-CoV isolate commonly used for in vitro experimentation accidentally infected a laboratory worker [10]. We sequenced both the originating laboratory isolate (SIN_WNV; see Table S1) and the viral sample directly from the patient's sputum (SIN0409; see Table S2) and found no sequence difference between the two viruses. This reconfirms that the mutation rate from a single point source of virus has a low mutation rate when expanded during human infection.

### Sequence variation filter

**Figure 1:**
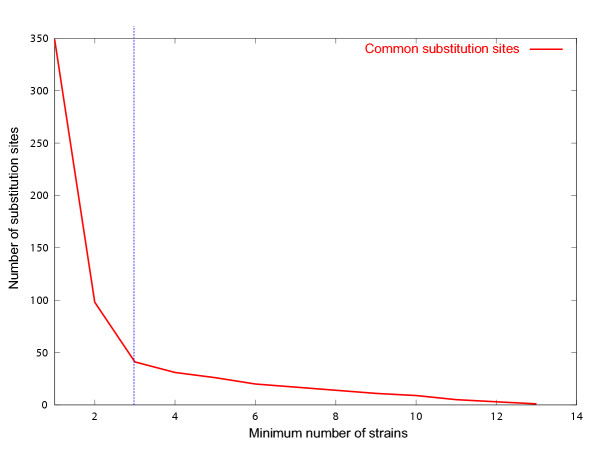
**Effects of applying mutation filter to number of substitutions observed. **To exclude nucleotide variations arising from sequencing error or other artifacts, we include only variations that are present in at least a number of different isolates. The graph shows an exponential decrease in the number of nucleotide variations detected with increasing filter stringency. The curve essentially flattens around 3, which corresponds to the filtering scheme that considers a sequence variation as a real mutation if it is shared by more than two isolates.

Inferring phylogenetic relationships between the known SARS-CoV isolates using existing public data has been problematic because of the potential for sequencing errors. Moreover, the rate of SARS-CoV mutation in culture was not previously known and was thought to be significant given the mutation rates in other RNA viruses. Our experiments provided information as to the potential causes and rates of sequence variations of the SARS-CoV in culture. Based on our Vero cells passages and human-to-Vero transition data, we estimated that, at most, one sequence variation from the original tissue virus can be accounted for by in vitro culture artifacts. The average base-calling error probability (as reported by PHREP [11]) of our sequences is about 7.5 × 10^-5^, or 2.25 errors per SARS-CoV genome. Accordingly, we suspected that sequencing errors in the reported SARS sequences would be approximately 1–2 bases per reported genome. We used this information to assess the true sequence variants reported in the public SARS sequence databases employing a "mutation filter" [5]. This mutation filter identifies a sequence variant as a probable mutation if it appeared in more than one isolate. Higher filter stringency can be applied by demanding a sequence variant to be present in two, three, or more isolates. A total of 54 isolates were analyzed, including 22 from Singapore. Our results show that the number of mutations appearing in only one isolate is high at 349 (see supplementary Table S3); however, those mutations present in more than two isolates are much lower and appeared relatively stable (Figure 1). Statistical analysis confirmed that the probability of finding any false mutation shared by more than two isolates out of 54 is very low (*p *≤ 2.2e - 4) as compared to the probability of finding a false mutation shared by more than one isolate (*p *≤ 0.19). These results are consistent with our error estimates as outlined above.

**Figure 2:**
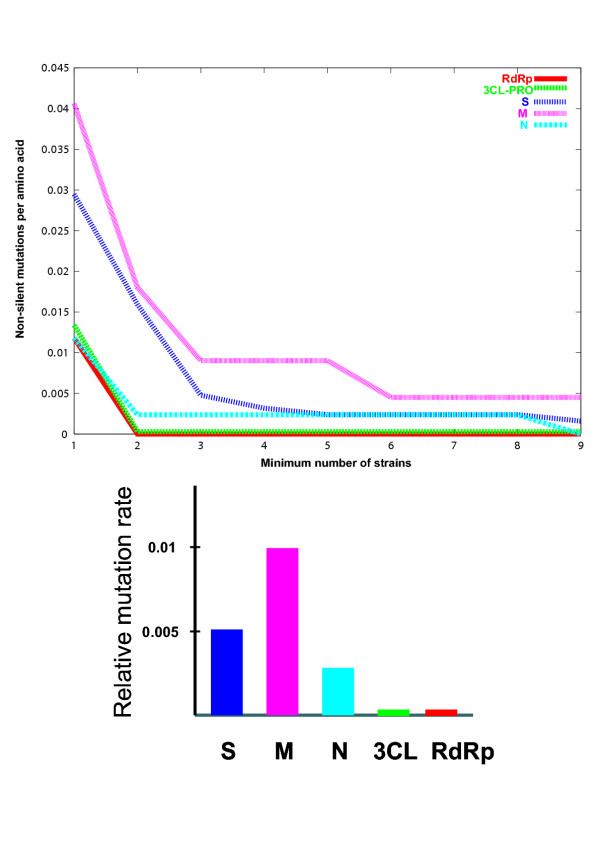
**Non-silent variations in five key ORFs. **Nucleotide variations that lie within a coding region might result in amino acid variations in the corresponding protein product. Such non-silent variations could play a significant role in determining the survivability of SARS-CoV variants. Application of the proposed variation filter reveals conservation of the polymerase (RdRp) and 3CLpro, which is consistent with previous studies of other Corona viruses.

We tested the biological validity of this approach by examining the mutational frequency of known genes in the SARS genome. Because of the importance of the 3CL protease and the polymerase for viral replication, we suspected that true non-synonymous mutations in the SARS-CoV present in clinical samples might be rare in these two ORFs in comparison to other structural genes such as those encoding the S, M, and N proteins. Without a mutation filter, sequence variations are commonly observed in the 3CL protease and the polymerase genes. However, when mutations are identified only as variants seen in two or more isolates, then no mutations are detected in the critical 3CL protease and polymerase genes, whereas mutations are noted in the S, M, and N genes regardless of the filter stringency (Figure 2). Therefore, we determined that the most effective mutation filter is presence of a sequence variant in more than two isolates.

### Molecular history of the viral isolates

**Figure 3:**
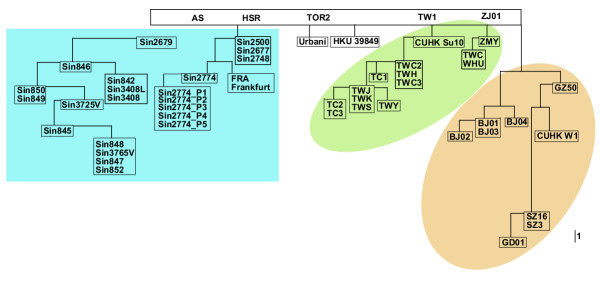
**Molecular relationship between 54 SARS-CoV genomes. **The phylogenetic tree reconstructed using PAUP* on nucleotide variations shared by more than two isolates. The tree was re-rooted on the earliest reported case, i.e. TOR2. Four major branches can be observed in the tree, each largely belonging to a certain geographical origin. Two of which encompass the Singapore cases, indicating the possibility of separate infection sources of the Singapore cases.

**Figure 4:**
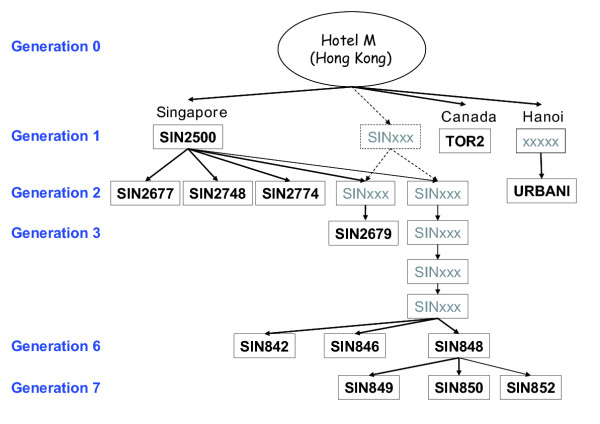
**Relations between the Singapore patients and others related to Hotel M, Hong Kong. **Contact tracing information of the Singapore patients and the two early reported SARS cases. Contact information revealed a single source of infection for Singapore patients, but molecular analysis, reported here and in (6), suggests the existence of an additional infection source (represented by dashed arrows and box) from the Hotel M case to Singapore.

Using this filter stringency, we assessed the phylogenetic relationship between all 54 isolates describing the recent SARS epidemic. TOR2, Urbani, SIN2500, HKU-39849, CUHK-Su10 formed the core of the early isolates. Employing these as "root", four major clusters appeared: two Singaporean branches, one Taiwan branch, and one North China branch (Figure 3). Validating these clusters, the two sequences from Germany, 'Frankfurt' and FRA, which grouped with the Singaporean branch, were actually derived from the Singaporean doctor who treated the patient SIN2774 and was later hospitalized in Frankfurt. Of the Singaporean cases, SIN2500, SIN2677, SIN2748, and SIN2774 formed one molecular sub-branch which matched with the contact tracing (Figure 4). The clinical contact tracing data was ambiguous as to the direct source of SIN2679's exposure. Intriguingly, however, SIN2679 was the root of a second sub-branch within Singapore that had its origins most probably from the Hotel M cluster. This suggested a potential direct infection of SIN2679 from a Hotel M source other than SIN2500. Using the same contact tracing information, we calculated the average mutation rate during human transmission to be about two mutations per human transmission.

### Estimation of the mutation rate of the SARS-CoV

We obtained the precise dates of symptom onset of 13 Singaporean cases (Table S1). Using the common mutations identified through application of the mutation filter, we employed the constant mutation rate model and estimated the mutation rate of the SARS-CoV during this recent epidemic. We estimated the mutation rate to be 0.1722 nucleotides per day, or 5.7 × 10^-6 ^nucleotide substitutions per site per day (adjusted R-square value of the fitted model = 0.4014). The rates for synonymous and non-synonymous mutations were equivalent at 2.5 × 10^-6 ^and 3.2 × 10^-6 ^nucleotide substitutions per site per day respectively. Using the Singapore isolates with known date of onset, and using the SZ3 and SZ16 genomes isolated from palm civet cat [12] as the putative "original" SARS-CoV that jumped from animal to human, we calculated the daily substitution rate to be 0.1303 nucleotides per day, or 4.3 × 10^-6 ^nucleotide substitutions per site per day, (adjusted R-square = 0.5880) and the estimated possible "date" of SZ3/SZ16 emergence was Oct 21, 2002. Overall, the mutation rate of SARS-CoV appears to be consistent with the reported rate of other viruses [13,14].

**Figure 5:**
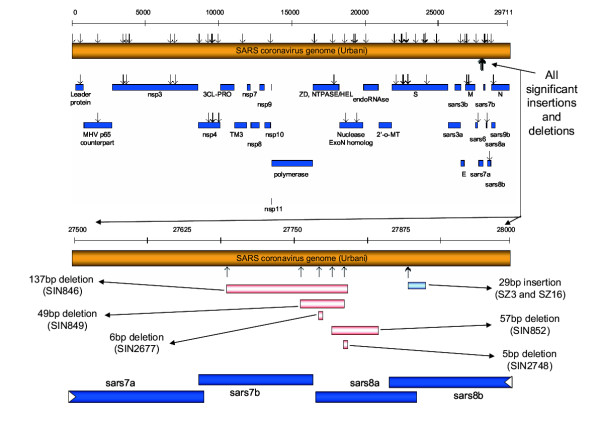
**Map of significant mutations, insertions and deletions in the SARS-CoV genome. **Map of the SARS genome, plotted with substitutions appearing in more than two isolates and other major insertion and deletion regions. Golden bars signify the SARS-CoV genome, with the approximate nucleotide positions shown in the scale on top of it. Arrows drawn on top of the genome indicate nucleotide mutations (i.e. variations shared by more than two isolates) observed in the SARS-CoV genome. Amino acid changes in SARS-CoV's proteins are reflected as arrows on top of the protein bars (blue bars). Significant multiple-nucleotide deletions (pink bar) and insertions (light-blue bar) were also observed (denoted as arrows under the genome) and appear to cluster around position 27000nt to 28000nt.

The mutational analysis also revealed 5 separate deletions and one insertion that distinguished the different isolates (Figure 5). Intriguingly, they all clustered within a short 200 bp region in the 3' end of the viral genome spanning putative ORFs sars 7b to sars 8b. Despite the overlapping nature of some of the deletions, there was no descendent relationship amongst them and the addition of the insertion/deletion information did not add to the clustering. Our assumption therefore is that this is a region of relative instability that is dispensable for viral replication.

## Conclusions

The focus of this investigation was to measure the mutational frequency and dynamics both in vitro and in vivo of the SARS-CoV. Our findings suggest that the overall SARS-CoV's rate of mutation in culture is low. Inoculation of Human SARS-CoV into Vero cell introduces, on the average, less than one nucleotide mutation. Subsequent culturing of SARS-CoV infected Vero cells induced less than one nucleotide mutation in the five consecutive Vero cell passages. No mutations were also observed during the infection of SARS-CoV cultured in Vero cell to human. This would be consistent with the notion that the SARS-CoV isolates from the patients that gave rise to the in vitro lines are well adapted for in vitro growth.

Our proposed mutation filter, which is based on these observations, seems to be stable and effective. Using this filter, reconstruction of molecular phylogenetic relations of the 54 SARS-CoV genomes revealed at least three major branches composed of cases related to Hotel M (Hong Kong), cases reported in North China, and cases found in Taiwan. Moreover, we show that these molecular sequence associations can be effectively used to more precisely reconstruct contact tracing. Our estimated the daily substitution rate of SARS-CoV to be 0.1722 nucleotides, or 5.7 × 10^-6 ^nucleotide substitutions per site, a mutation rate similar to other RNA viruses [13,14]. Taking the SARS-CoV isolated from palm civet cat as the putative originating SARS-CoV, our calculations suggest that the earliest possible date for SARS emergence is predicted to be Oct 21, 2002. During the final preparation of this manuscript two reports were published addressing the mutation rate of the SARS-CoV in human populations. Yeh *et. al. *[15], examining Taiwanese SARS samples estimated the CoV mutation rate to be about 1.83 × 10^-6 ^nucleotides per site per day. The Chinese SARS Molecular Epidemiology Consortium [16], examining a larger number of viral isolates, recently determined the mutation rate to be 8.26 × 10^-6 ^nucleotides per site per day using samples from China. These estimates were very close to ours. In addition, The Chinese Consortium [16] projected the time of emergence of the SARS CoV epidemic to be November 2002. The remarkable consensus of these three studies using different patient populations on the mutational dynamics of the SARS CoV suggests that these results are bona fide.

## Competing interests

None declared.

## Authors' contributions

VBV and ETL performed most of the data analysis and prepared the draft manuscript. STSY and LAE provided SARS-CoV samples from patients and Vero cells. KFT and OEE provided continue SARS-CoV containing Vero cell passage samples. YR and LWS designed the experiments and coordinated sample acquiring and the viral genome sequencing. CLW and TZ generated all DNA sequence data. WHL processed all DNA sequence and assembled the viral genomes. JL verified all sequence variations by conducting MALDI-TOF mass spectrometry analysis. PK assessed the effects of nucleotide variations to ORFs and their proteins. VBV and PML carried out the mathematical and statistical analysis.

## Pre-publication history

The pre-publication history for this paper can be accessed here:



## Supplementary Material

Additional File 1 Table S1 List of the sequences used in the analysis. This list is available at http://giscompute.gis.a-star.edu.sg/sars_mut_dyn/ Click here for file

Additional File 2 Table S2 Single nucleotide variations (SNVs) detected initially in capillary sequencing (second row) and subsequently confirmed by MALDI-TOF MS-based genotyping (first row). This table is available at http://giscompute.gis.a-star.edu.sg/sars_mut_dyn/ Click here for file

Additional File 3 Table S3 Complete list of single nucleotide variations (SNVs) observed in the 54 SARS-CoV isolates. The Singapore sequences used were all based on capillary sequencing. This data is available at http://giscompute.gis.a-star.edu.sg/sars_mut_dyn/ Click here for file

## Figures and Tables

**Table 1:** Table 1: Single nucleotide heterogeneity (SNH) observed in the six passages of Vero cells culture. Along the six Vero cell passages, nucleotide heterogeneity was observed (initially through capillary sequencing and confirmed using MassARRAY genotyping) at nucleotide position 18356. Presence of single nucleotide heterogeneities (SNHs) indicates coexistence of multiple SARS-CoV isolate in the Vero cell culture.

**Genomic Location (Based on SIN2774)**	**SIN2774_P1**	**SIN2774_P2**	**SIN2774_P3**	**SIN2774_P4**	**SIN2774_P5**	**ORF**
18356	A/G	A/G	A/G	A/G	A/G	135aa of nuclease ExoN homolog *[R/G]*

**Table 2:** Quasispecies fluctuations and mutations during the transition from human tissue to Vero cell culture. Nucleotide variations observed between primary human tissue isolates and their respective subsequent Vero cell culture. Both quasispecies selection (SIN3275V → SIN849M) and new emergence (AS → HSR1) are observed during the transmission of SARS-CoV from human tissue sample into Vero cell culture.

**Source of viral sequence: Human Tissue**	**Source of viral sequence: Passage to Vero cell culture**	**Nucleotide Heterogeneity**	**Position (Urbani)**	**ORF (based on NC_004718.3)**
Sin3408L	Sin842M	-	-	-
Sin3725V	Sin849M	Y [t/C] → T	548	95aa of Leader protein (I, T → I)
		Y [T/C] → C	13347	131aa of NSP10 (SILENT)
Sin3765V	Sin852M	-	-	-
AS	HSR 1	G → R [G/A]	27254	637aa of sars6 (D → D, N)

**Table 3:** Nucleotide variations during the transition from Vero cell culture to human. No nucleotide variations were observed between isolate from laboratory-acquired SARS patient and its infection source.

**Source of viral sequence: Vero cell culture**	**Source of viral sequence: Passage to Human Tissue**	**Number of stable nucleotide substitutions**
SinWNV	Sin0409 (sputum)	0
